# Translating and Testing a Digital Game Promoting Vegetable Consumption in Young Children: Usability Study

**DOI:** 10.2196/43843

**Published:** 2023-10-03

**Authors:** Sophie Bucher Della Torre, Marlene Lages, Sara S Dias, Maria P Guarino, Cátia Braga-Pontes

**Affiliations:** 1 Geneva School of Health Sciences HES-SO University of Applied Sciences and Arts Western Switzerland Carouge Switzerland; 2 ciTechCare- Center for Innovative Care and Health Technology Polytechnic of Leiria Leiria Portugal; 3 School of Health Sciences Polytechnic of Leiria Leiria Portugal

**Keywords:** vegetable, food preference, serious games, video game, children, child, pediatric, obesity prevention, pilot study, gaming, educational game, nutrition, diet, healthy eating, food consumption, food intake

## Abstract

**Background:**

Promoting healthy eating in children is key to preventing chronic diseases, and vegetable consumption is notably lower than recommended in this population. Among the interventions tested, gamification has shown promise in promoting familiarization, increasing knowledge, and potentially increasing vegetable intake.

**Objective:**

This pilot study aimed first to translate the digital game “Veggies4myHeart” into French and to assess its influence on young children’s preferences and willingness to taste vegetables when combined with repeated tasting and education. We also aimed to investigate the acceptability and applicability of the game in 2 classrooms.

**Methods:**

During 5 consecutive weekly sessions, children from 2 elementary classes played the digital game consisting of 5 mini games on different vegetables (lettuce, carrot, red cabbage, cucumber, and tomato) in pairs for 10-15 minutes. In addition, they discussed one of the vegetables and tasted the 5 vegetables in each session. Pretest and posttest food preferences and willingness to taste the vegetables were compared. Teachers participated in a semistructured interview.

**Results:**

A total of 45 children aged 5 to 6 years tested the French version of the digital game. The children’s declared food preferences were already high for carrot, cucumber, and tomato, with scores higher than 4 out of a maximum of 5. The scores did not change significantly after the intervention, except for red cabbage (pretest: mean 2.52, SD 1.49; posttest: mean 3.29, SD 1.67; *P*=.006) and a composite score (pretest: mean 3.76, SD 1.06; posttest: mean 4.05, SD 1.03; *P*=*.*001). Before the intervention, 18 (44%), 30 (73%), 16 (39%), 29 (71%), and 26 (63%) children out of 41 were willing to taste lettuce, carrot, red cabbage, cucumber, and tomato, respectively. After the intervention, no significant statistical differences were observed, with 23 (51%), 36 (80%), 24 (53%), 33 (73%), and 29 (64%) children out of 45 willing to taste lettuce, carrot, red cabbage, cucumber, and tomato, respectively. Teachers supported this tool combined with repeated tasting and education and highlighted facilitators and barriers that should be anticipated to improve implementation in schools.

**Conclusions:**

In this study, we translated an existing digital game applicable and acceptable to both children and teachers. A larger study is warranted to confirm the effectiveness of interventions using the digital game to promote vegetable preference, willingness to taste, and intake.

## Introduction

### Vegetable Consumption in Children

Healthy eating is recognized as a cornerstone of health promotion [1], yet there is a persistent gap that exists between dietary recommendations and actual intake [2]. This is particularly true for children, and especially with regard to fruit and vegetable consumption [3-5]. Establishing healthy eating habits during infancy and childhood is a widely shared public health goal [6], and the promotion of fruits and vegetables is often at the heart of such programs [7]. In fact, fruit and vegetable consumption is a protective factor for many health conditions [8]; however, children often begin to reject these foods during the developmental phase of neophobia [9]. Food neophobia is defined as the fear of eating new foods and appears around the age of 2 years, with a paroxysm between the ages of 2 and 6 years [10]. During this time, children tend to reject any new food or food presented in a novel way; this is especially true for vegetables, which is often explained by their low energy density and strong taste [10]. Evidence shows that the effects of food neophobia can be limited by increasing familiarity with the food and promoting exposure, associative learning, and tasting [11,12].

### Serious Games to Promote Vegetable Intake

Gamification is an interesting new approach to engage children with vegetables in an attractive way. Moreover, this tool may be particularly suitable for highly neophobic children, as it works through visual exposure [13]. Some traditional games, such as memory or board games, have been shown to have a positive influence on eating behaviors [14,15], and the ability of digital games to improve health and food habits has also been tested, taking advantage of their ability to promote interactivity, provide fun, and attract attention [16]. The “Veggies4myHeart” digital game was developed in Portugal and consists of 5 mini games, each featuring a vegetable superhero. It was successfully tested with children aged 3-6 years and, when combined with exposure through vegetable tasting, led to an increase in vegetable consumption [17].

### Challenges in Developing and Using Serious Games

Developing digital games is time-consuming and resource-intensive [16]. Using existing games is a solution to save resources; however, whether cultural specificities exist should be clarified before translating and using such games on a large scale. The question of how to use digital games more effectively also remains open. A digital game can be used as a stand-alone tool or integrated into a larger program and combined with other features.

### Objectives

The aim of this pilot study was to translate the digital game Veggies4myHeart into French and to assess its influence on the preferences and willingness to taste vegetables of young Swiss children in combination with repeated tasting and education. As a second objective, we investigated teachers’ opinions about the game and its acceptability and applicability in two classrooms.

## Methods

### Participants

This pilot study involved 45 children from two classes of an elementary school in the French-speaking part of Switzerland and their teachers. The children were between 5 and 6 years old and included 19 boys and 26 girls. The children from the two classes received the same intervention. The two teachers volunteered to participate in the study.

### Ethical Considerations

Parents were informed about the intervention and could request that their child not participate in the study. No personal data were collected, so this pilot study did not fall under the Swiss Federal Human Research Act. However, it was submitted to and approved by the Service of Research in Education in Geneva, Switzerland (research number 584).

### Veggies4myHeart Digital Game and Translation

The Veggies4myHeart digital game was developed by a team of researchers at the Polytechnic of Leiria in Portugal [17]. The digital game was designed for preschool children aged 3-6 years and consists of 5 mini games, each related to one of the following vegetables: carrot, tomato, lettuce, cucumber, and red cabbage. For each vegetable, an introduction presents the vegetable, its characteristics, and health benefits and explains the goal of the task in the game.

The game setting is a farm. Children, through the vegetable superheroes, are involved in watering the fields, protecting the vegetables from pests, or picking the vegetables. In each mini game, children must race against the clock to collect as many points as possible, winning medallions at each step. Children can also customize their superheroes.

The Veggies4myHeart video game was developed to support the personal factors of motivation and knowledge that are necessary to improve the targeted behavior. Indeed, in a model based on social cognitive theory, Reynolds et al [18] show how environmental factors (availability, modeling, and nutrition education) and personal factors of motivation and knowledge can improve the specific behavior of fruit and vegetable consumption in young children. Specifically, the game enhances the following important determinants of vegetable consumption: (1) perceived self-efficacy through positive experiences with vegetables, persuasion, and by convincing the children of their ability to eat vegetables in different situations; (2) outcome expectations through age-appropriate persuasive communication and game stories that emphasize the positive effects of eating vegetables; and (3) food preferences by increasing familiarity with the vegetables. Knowledge about vegetables is also increased through the various messages delivered throughout the game. In addition, several known mechanisms involved in learning to eat vegetables underlie our intervention [19]. For example, the digital game allows nontaste exposure to vegetables. Previous studies have shown that it is possible to increase familiarity in the absence of real food through picture books, storybooks, and digital media [13,20]. Associating images of vegetables with a narrative or a character enhances the playful element of the learning experience and encourages a willingness to taste by enabling a positive affective experience during exposure [19]. Rewards, ranging from social praise to small nonfood gifts such as stickers, are another well-known strategy to increase learning and have been used to facilitate children’s intake of fruits and vegetables and promote liking [21]. In digital games such as Veggies4myHeart, rewards are used differently: as participants progress through the game, they earn achievements, prizes, or rewards that encourage learning and increase extrinsic motivation to play the game [22,23].

**Figure 1:**
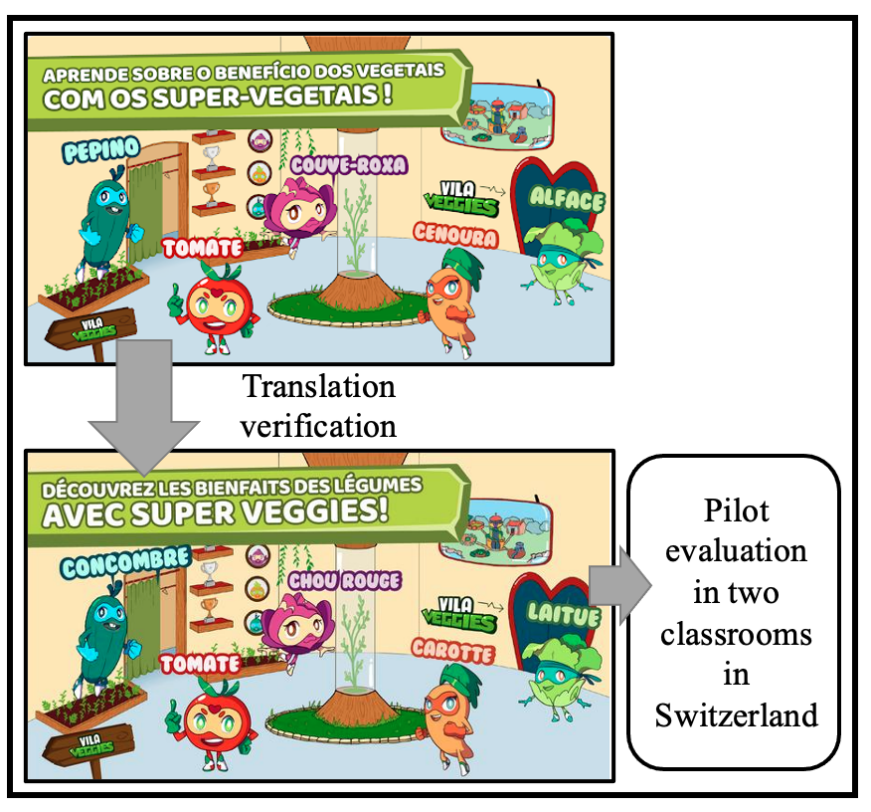
Steps to translate Veggies4myHeart from Portuguese into French and to test it in Switzerland.

The digital game was originally developed in Portuguese. A Portuguese French teacher translated the scripts into French, a Swiss researcher verified the French script, and two children recorded the texts in French, as illustrated in Figure 1.

### Procedures of the 5 Sessions

The research team visited each class for the intervention once a week for 5 consecutive weeks. Each session focused on one vegetable: carrot, red cabbage, lettuce, tomato, and then cucumber. The study took place in May and June 2021.

At the beginning of each session, the dietitian introduced the vegetable of the day and asked the children if they knew how to prepare or cook it. The characteristics and benefits of the vegetable were discussed. This short educational session was designed to complement the digital game.

The children then got into pairs and played the Veggies4myHeart digital game described below on an iPad-type tablet. Each child played the mini game related to the vegetable of the day for 10-15 minutes.

During each session, children were invited to taste the 5 vegetables presented raw and plain in small pieces and the investigator recorded the types of vegetables tasted. Repeated exposure was included in the intervention because it is a very simple yet powerful strategy to promote vegetable intake, as shown in a meta-analysis published in 2018 [24]. The authors found that intake was positively associated with the amount of taste exposure and was greater for unfamiliar vegetables and when they were presented in plain form [24]. Repeated exposure allows children to increase their familiarity with unknown vegetables and to learn about their safety, resulting in increased acceptance and liking [19].

At the end of the intervention, children received a booklet with recipes using the 5 vegetables in the game and information for parents on how to download the game for free if they wished. Throughout the whole process, the children were unaware of the objectives of the study.

### Pre-Evaluation and Postevaluation of Preferences and Willingness to Taste

Two identical standardized assessment sessions were conducted by a team of dietitians. These sessions took place 1 week before the first session and 1 week after the last session. The assessment included an evaluation of the preferences of 10 food items, including 8 fruits and vegetables, and an assessment of the willingness to taste the 5 vegetables in the game as a way of evaluating behavioral neophobia [25].

To evaluate preferences, each child was asked to indicate his or her preference for each of the 10 photographed foods using a visual rating scale of 5 smileys (5: like it a lot; 4: rather like it; 3: neither like or dislike it; 2: rather dislike it; 1: dislike it a lot). The document used for this evaluation is provided in Multimedia Appendix 1. To assess the willingness to taste, each child was invited to taste the 5 vegetables from the digital game (ie, carrot, tomato, lettuce, cucumber, and red cabbage). The 5 vegetables were cut into small pieces and presented simultaneously on a table in front of the child, with a similar level of accessibility. The number of different vegetables tasted was counted.

Throughout the assessment, the children were assessed individually, and the dietitian remained neutral throughout the assessment (no help, praise, or encouragement). With the help of the teachers, a code corresponding to the child’s number on the class list was used to identify the children. Thus, the investigators did not collect any personal data on the children.

### Assessment of Acceptability and Applicability

To answer our second objective, the investigators observed and took notes on the process of each session. After the 5 sessions, they collected the teachers’ opinions on the usefulness and acceptability of the digital game, potential adaptations, and the facilitators and barriers to its use in schools. Both teachers participated in a common semistructured interview based on open-ended questions (Multimedia Appendix 2). The interview was recorded and the teachers signed a consent form.

### Data Analysis

Descriptive statistics are reported as means and SD, medians and IQR, frequencies, and percentages. Pre-post comparisons were performed using the Wilcoxon test for paired samples due to a lack of normal distribution. *P*<.05 was considered statistically significant. All analyses were performed using SPSS Statistics 27 (IBM Corp).

Regarding the teacher interviews, one researcher analyzed the data using a qualitative descriptive approach based on notes and recordings. The researcher identified dominant and relevant themes associated with the data and highlighted conceptual similarities and differences between the two teachers. Two other researchers reviewed the analysis and checked the themes for reliability. Themes were then synthesized, classified, and analyzed based on the interview grid and predefined research questions.

## Results

### Translation and Implementation

The Veggies4myHeart digital game, originally developed and accessible in Portuguese, was successfully translated into French. The narrative of the game and the messages about the 5 vegetables did not need to be changed, and no cultural adaptation was required. The main adaptation in game use related to the seasons in which the 5 vegetables are grown locally in Switzerland. Children and teachers did not report any problem with comprehension. The intervention was implemented as planned; however, a 45-minute period was necessary when taking into account the time needed for the children to settle into the classroom, wash their hands, and socialize with the dietitian.

### Pre-Evaluation and Postevaluation of Preferences and Willingness to Taste

The pretest evaluation highlighted that some vegetables in the game already scored high in the children’s food preferences, especially carrots. On a scale of 1-5 (5 being “I like it a lot”), the mean rating was >4 for carrots, cucumber, and tomato, as well as for apple, which was not part of the video game. The liking of carrots further improved by another 7%, but the greatest increase in preference was for red cabbage, which increased significantly by 31% to a mean score of 3.29 (SD 1.67; *P*=.006), as shown in Table 1. When combining the scores of the 5 vegetables in the game into a composite score, the children’s liking improved post intervention. The preference for fruits and vegetables not present in the game did not change significantly in pre-post comparisons. Before the intervention, the mean score was 3.88 (SD 1.54) for apricots, 2.12 (SD 1.48) for fennel, and 4.31 (SD 1.35) for apples, and after the intervention, the mean scores were 4.09 (SD 1.31), 2.11 (SD 1.56), and 4.51 (SD 1.12), respectively.

A similar progression was seen in the willingness to taste task, with 24 (53%) children willing to taste red cabbage after the intervention compared to 16 (39%) before. However, none of the differences in willingness to taste were statistically significant (Table 2).

**Table 1:** Preintervention and postintervention mean score of preference of the five vegetables in the video game.^a^

	Pretest (n=42), mean (SD)	Posttest (n=45), mean (SD)	*P* value^b^
Lettuce	3.90 (1.46)	4.02 (1.47)	.37
Carrot	4.19 (1.40)	4.49 (0.99)	.16
Red cabbage	2.52 (1.49)	3.29 (1.67)	.006
Cucumber	4.10 (1.54)	4.31 (1.40)	.12
Tomato	4.07 (1.50)	4.13 (1.50)	.12
Composite score	3.76 (1.06)	4.05 (1.03)	.001

^a^Maximum score=5.

^b^Wilcoxon test for paired samples.

**Table 2:** Number and proportion of children who tasted each vegetable before and after the intervention.

	Pretest (n=41), n (%)	Posttest (n=45), n (%)	*P* value^a^
Lettuce	18 (44)	23 (51)	.25
Carrot	30 (73)	36 (80)	.23
Red cabbage	16 (39)	24 (53)	.09
Cucumber	29 (71)	33 (73)	.39
Tomato	26 (63)	29 (64)	.46

^a^Wilcoxon test for paired samples.

### Teachers’ Opinion on Acceptability and Applicability

Two teachers commented on the usefulness and acceptability of the digital game, the potential adaptations needed, and the facilitators and barriers to its use in schools.

Both teachers rated the game very positively and found it appropriate for children aged 4-6 years. They observed that the children were very interested and engaged in the activity. The teachers appreciated the progressive nature of the game, which allowed the children to start with easy levels and then move on to more complex games. In the teachers’ view, the game helped to develop knowledge of the names of vegetables and familiarity with them. By remembering what they had to do in the game, the children were able to remember some functions of the vegetables (eg, watering the garden as an illustration of hydration).

The teachers appreciated that the children played in pairs, as this favored interesting interactions between the children. The initial 15 minutes of play was felt to be too long; 10 minutes per child was sufficient for the final sessions. In addition to the game, the teachers felt it was important to offer a tasting and a discussion about the different ways of consuming the vegetable in each session to give a concrete aspect to the activity.

To promote and facilitate the integration of the information, the teachers recommended systematically repeating the messages of the game during the conclusion of the activity following the game sequence. They also suggested developing visual material, such as pictograms for each vegetable or a laminated map of the game to facilitate navigation in the game.

Regarding the implementation of the activity in the classroom, the teachers stated that they could manage the sequence on their own, but this would require having tablets readily available.

## Discussion

### Principal Findings

In this study, a Portuguese digital game consisting of 5 mini games about vegetables was successfully translated into French and tested in two classrooms with children aged 5-6 years. No cultural adaptation was necessary, and the children played the digital game without any difficulties. The teachers confirmed their interest and the feasibility of integrating such a game into their teaching. In addition to the game, the children received information about the vegetables in the game and had the opportunity to taste them repeatedly. However, although the pre-post comparison of preferences and willingness to taste vegetables showed some promising trends, few differences were statistically significant.

### Comparison With Prior Work

In the literature, eHealth interventions to promote fruit and vegetable intake consistently produce small but significant improvements. In a meta-analysis that included 19 studies (only 4 of which were in children), all showed a small effect size favoring eHealth interventions [26]. Tailored interventions and those using at least 7 behavior change techniques were the most effective [26]. Given the many factors that influence vegetable consumption, it is expected that no single tool or strategy will work for all children. Multicomponent interventions, on the other hand, combine several techniques, increasing the likelihood that all children will benefit from one strategy or another [19]. Interestingly, brief digital interventions can already be effective, although repeated exposure strengthens the effects [27]. The first study to test the Veggies4myHeart video game was conducted in Portugal. In a preschool, the video game combined with repeated tasting was compared with the use of a storybook and repeated tasting with or without sticker rewards and a control group [17]. After the intervention and at a 6-month follow-up, children in all groups, including the control group, had increased their vegetable consumption. In another study conducted in Taiwan with children aged 5-6 years, a 4-week intervention based on a computer game called “Healthy Rat King” resulted in increased nutritional knowledge among participants, but no differences in junk food consumption were observed compared to the control group [28]. Using a mobile app based on repeated visual exposure, modeling, and rewards, Farrow et al [29] showed an increase in the consumption and liking of vegetables displayed in the app compared to control group. Digital games raise a lot of interest and expectations to promote healthy eating in children, but studies evaluating them, including ours, often lack sufficient sample sizes and appropriate designs to demonstrate their effectiveness and to describe the mechanisms involved [30]. In addition, developing a digital game is expensive and requires a multidisciplinary team of experienced and skilled members [16]; therefore, to conserve resources, translating existing games is a rational and attractive option. In a previous study, an American digital game designed to reduce the risk of obesity and type 2 diabetes was translated and culturally adapted for Hong Kong Chinese children. As in our study, their evaluation, based on a survey and individual interviews, confirmed the acceptability and applicability of the game in the new context [31,32].

### Digital Games as Prevention Tools

Serious games expand the range of learning tools available to promote balanced nutrition. We know that, in addition to experiential learning, playful activities enhance children’s learning. Digital games appear to be an attractive complementary tool to promote vegetable intake while maintaining a balance between education and play and enjoyment, as an overemphasis on education risks reducing learning [16,19]. Indeed, both enjoyment and user experience have been shown to be positively correlated with learning, as measured by increased knowledge in children [33].

In addition to the game itself, implementation is a critical consideration for effective health promotion interventions. In this study, dietitians implemented the intervention. To scale up and sustain this type of intervention, teachers should be able to implement it by themselves. Following our experience, the two teachers confirmed that they thought the digital game could be used as an additional learning tool, as long as they had support for the logistical aspects, for example, from the school administration. In this respect, our game is easily accessible as a free application on common platforms. To further support teachers, some additional material would be useful, such as a teacher’s guide with background information on the overall benefits of vegetables and the specifics of each vegetable, prompts and visuals for class discussions on each vegetable, and recipes. Because the Veggies4myHeart game is freely available, children can continue to play the game at home, potentially maintaining or increasing the impact of the intervention.

### Strengths and Limitations

Despite the encouraging results, this pilot study has several limitations. First, the sample size was small and the study lacked a control group to isolate the effects of the intervention. Second, the design did not allow us to analyze the effect of the digital game separately from the effects of the discussions and tastings that were conducted concurrently, nor to identify which game elements were the more effective. Third, we did not measure the children’s eating traits, such as fussiness. These factors should be taken into consideration in future analyses. However, based on a quantitative and qualitative evaluation, this study provides useful information for the development and implementation of future interventions that include a digital game to promote vegetable consumption in young children.

### Future Directions

Future studies should include a larger sample size and a control group, ideally designed with multiple arms to allow a comparison between elements of combined interventions. Moreover, future samples should include children who do not especially like the vegetables to avoid or limit the ceiling effects observed in our study. Could digital games support food literacy through the development of functional and relational skills? This would be interesting to assess, as a digital game may promote basic nutrition knowledge and a healthy and positive relationship with food and encourage the experience of new and different foods, which are all important elements of food literacy [34]. More generally, larger studies including greater sample sizes are needed to assess the actual impact of digital games and to reach statistical significance.

### Conclusions

Digital games are a useful and engaging tool for promoting vegetables to young children. In this study, an existing video game was translated into French and paired with repeated tasting and education to combine multiple strategies and increase the chance of reaching every child. Our experience shows that the digital game was easy for teachers to use in younger grades, but the logistical aspects should be very well prepared. Pre-post comparisons of preferences and willingness to taste vegetables showed some promising trends; however, confirmation with larger samples is warranted.

Using existing games and collaborating to translate them is a promising way to maximize development investments and increase the use and reach of such games. Future games should focus on unfamiliar vegetables and include in-game progression.

## Appendix

Multimedia Appendix 1 Questionnaire used to assess children’s food preferences.

Multimedia Appendix 2 English translation of the questions asked to the teachers regarding their opinion on the video game and the possibilities to use it in the classroom.

## Data Availability

The data sets generated or analyzed during this study are available from the corresponding author on reasonable request.
