# Supplementary material for: Translating and Testing a Digital Game Promoting Vegetable Consumption in Young Children: Usability Study
Source: JMIR Serious Games. 2023 Oct 3;11:e43843. doi: 10.2196/43843 (PMC10582818; doi:10.2196/43843)
Supplement: Multimedia Appendix 1 [file games_v11i1e43843_app1.pdf]

Questionnaire used to assess children's food preferences

Veggies4myHeart

Évaluation d'un jeu vidéo visant à promouvoir la consommation de légumes chez des jeunes enfants

Date: ____/____/________	**Feuille d'évaluation des préférences alimentaires des enfants**	Code de l'enfant (à compléter par le chercheur)

1. Aimes-tu les aliments photographiés ci-dessous ?

1 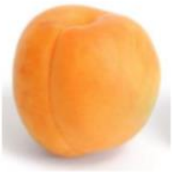	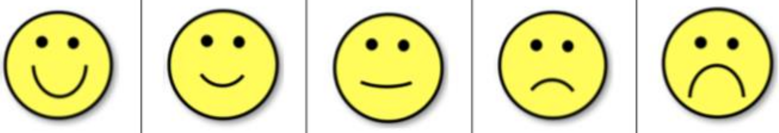
2 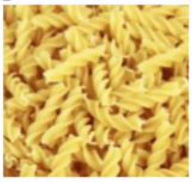	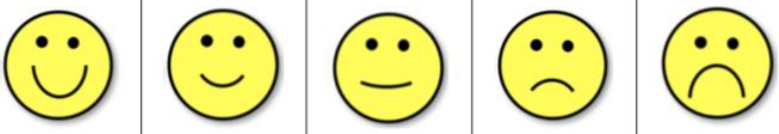
3 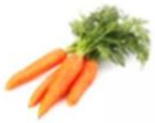	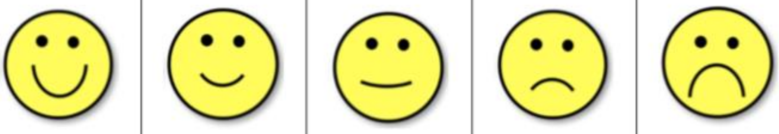
4 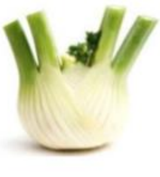	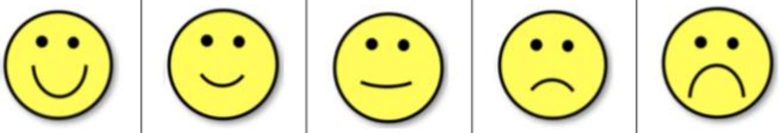
5 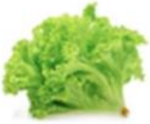	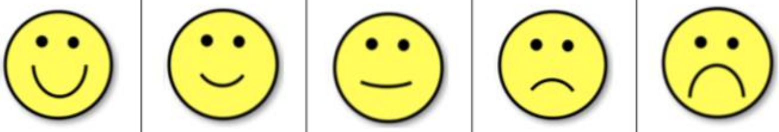

Haute école de santé Genève
Filière Nutrition et diététique

Veggies4myHeart
Évaluation d'un jeu vidéo visant à promouvoir la consommation de légumes chez des jeunes enfants

6 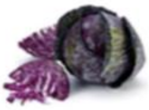					
7 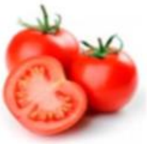					
8 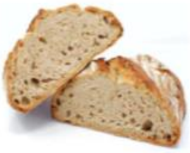					
9 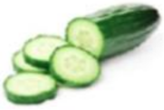					
10 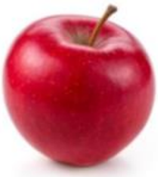					
