# Supplementary material for: Translating and Testing a Digital Game Promoting Vegetable Consumption in Young Children: Usability Study
Source: JMIR Serious Games. 2023 Oct 3;11:e43843. doi: 10.2196/43843 (PMC10582818; doi:10.2196/43843)
Supplement: Multimedia Appendix 2 [file games_v11i1e43843_app2.pdf]

English translation of the questions asked to the teachers regarding their opinion on the video game and the possibilities to use it in the classroom.

Veggies4myHeart: interview guide

a. Following this experience, what do you think of this video game and its use in class?
b. In your opinion, is it a useful tool for developing knowledge of vegetables and promoting their consumption?
c. Is the game age appropriate? Do you think it could be used in 1th, 2nd, 3rd, 4th grade?
d. From your teacher's point of view, what is the acceptability to use this game in the classroom?
e. Do you see any potential needed adaptations to the game?
f. What factors could facilitate or are barriers to its use in school?
